# Taxonomy and biology of two seed-parasitic gracillariid moths (Lepidoptera, Gracillariidae), with description of a new species

**DOI:** 10.3897/zookeys.83.783

**Published:** 2011-02-25

**Authors:** Bingbing Hu, Shuxia Wang, Jing Zhang, Houhun Li

**Affiliations:** College of Life Sciences, Nankai University, Tianjin 300071, China

**Keywords:** Lepidoptera, Gracillariidae, *Conopomorpha*, *Epicephala*, *Flueggea suffruticosa*, new species, biology

## Abstract

A new species and new record of gracillariid moths from China are reported: Conopomorpha flueggella Li, **sp. n.** and *Epicephala relictella* Kuznetzov, 1979. Specimens were collected on flowers or leaves of Flueggea suffruticosa (Pall.) Baill. (Euphorbiaceae) at night, and reared from fruits in captivity. Larvae of both species feed on the seeds of Flueggea suffruticosa, but they can be differentiated externally by the position of the red pattern on the thorax and abdomen. Morphology of the eggs, larvae, pupae and the life history of the two species are described and compared. Images of the life history and figures of the genital structures are provided.

## Introduction

Most species of Gracillariidae are leaf-miners, although some are stem-, fruit- or peel-miners or feed on flower buds (Fletcher 1920, Huang et al. 1997; Vargas and Landry 2005, Grafton-Cardwell et al. 2008, Kawahara et al. 2009). An obligate pollination mutualism exists between Epicephala moths and Euphorbiaceae (or Phyllanthaceae) trees. The seed-parasitic habit of Epicephala, as in the pollination mutualisms of fig wasps, yucca moths and senita moths,is unique in Gracillariidae (Riley 1892, Fleming and Holland 1998, Bai and Li 2008, Weiblen 2002, Kato et al. 2003, Pellmyr 2003, Kawakita and Kato 2004a and b, Kawakita 2010). Currently, the biology and hostplants of most Epicephala species remain unknown according to our recent study. Previous studies in the Euphorbiaceae–Epicephala mutualism have revealed a high degree of specificity between pollinating moths and plants, although the relationships between hostplants and insects are not always an exact one-to-one relationship (Kawakita and Kato 2006). We found a high degree of diversity in the biology of adults and larvae, which enables us to understand more about the obligate pollination mutualism between the Epicephala moths and the Euphorbiaceae trees.

In this paper, we describe the morphology and biology of adults and larvae of Conopomorpha flueggella Li, sp. n. and Epicephala relictella Kuznetzov, 1979. Both species are seed–parasites of Flueggea suffruticosa in the Baxian Mountain State Nature Reserves in Tianjin, China. The hostplant of Epicephala relictella is here recorded for the first time, and the biology of the two gracillariid species is described and compared. Images of the adults and genitalia are provided.

## Material and methods

**Figures 1–4.**
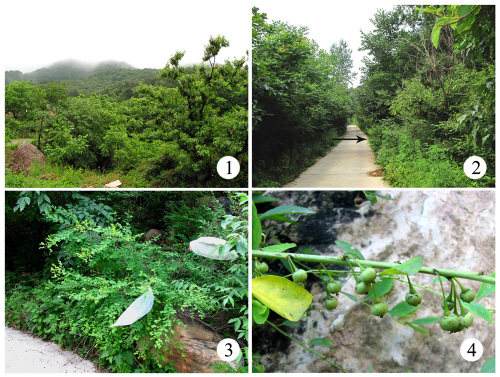
Habitats and host plants of two gracillariidspecies in Baxian Mountain State Nature Reserves. **1** general habitat **2** habitat of Flueggea suffruticosa, arrow pointing to host plant **3** female individual of Flueggea suffruticosa **4** fruits of Flueggea suffruticosa.

Field studies were conducted from 2007 to 2009 in the Baxian Mountain State Nature Reserves (40°7'24"–40°13'53"N, 117°30'35"–117°36'24"E) in Tianjin, China (Fig. 1), at an altitude ranging from 500 to 800 m. The area covers 5360 hm2, with 1583 hm2 as the core region. It is characteristic of warm temperate deciduous broad-leaved forest, and belongs to the warm and humid continental monsoon climate. The annual average rainfall amounts to 968.5 mm, and the annual average temperature is 8–10 °C (Li et al. 2009).

Flueggea suffruticosa (Pall.) Baill. (Fig. 3) occurs in scrubby slopes, forest margins and at road sides (Fig. 2) at an altitude of 500 to 2500 m. It is distributed in China (except in Gansu, Qinghai, Xinjiang and Tibet), Japan, Korea, Mongolia and Russia (Li 1994). Flueggea suffruticosa (Pall.) Baill. forms typically 1–3 m tall shrubs, is dioecious, and the inflorescences are axillary and cymose. The male flowers have 3–18 clusters, 5 sepals, free filaments, and 5 stamens. The female flowers have 3 styles, erect to spreading horizontally, free or connate at base, and bifid; the ovary is 3-celled, each cell having 2 ovules. The fruit is an oblate capsule, reddish brown when ripe (Fig. 4). The flowering period lasts from May to August, and the fruiting period from June to November in Baxian Mountain.

The biology of Conopomorpha flueggella Li, sp. n. and Epicephala relictella Kuznetzov was observed and studied during August–October 2007 and May–October of 2008 and 2009. Life history observations were made during flowering and fruiting seasons. The developing and mature fruits were collected from different individuals and dissected to examine the feeding habit with a light microscope. In addition, the developing fruits were collected in a cylindrical box (10 cm × 10 cm2) to rear mature larvae and braconid wasps, and the behaviors of the mature larvae were observed.

Specimens examined in this study were collected on flowers or leaves of Flueggea suffruticosa at night, and reared from fruits in captivity, and a few specimens were collected by using light traps. Genitalia dissection and mounting follow Li and Zheng (1996). Photographs of Flueggea suffruticosa and moths were taken primarily in the field using Canon G10 and Canon S3 IS digital cameras. Photographs of adult specimens were taken with a Nikon D300 digital camera. Dissections of genitalia were conducted under an Olympus SZ11 stereo zoom microscope. Figures of genitalia were prepared using an Olympus C-7070 digital camera attached to an Olympus BX51 microscope.

The type specimens are deposited in the Insect Collection, College of Life Sciences, Nankai University, Tianjin, China.

## Taxonomic history

Meyrick (1885) established the genus Conopomorpha based on the type species Conopomorpha cyanospila Meyrick, 1885. The wing pattern suggests it may be closely related to Epicephala Meyrick, 1880, but the complete separation of the sacculus and costa as well as the simple ovipositor distinguishes Conopomorpha from Epicephala.

Conopomorpha currently consists of 13 species worldwide: eight species in the Australian Region, three in the Oriental and Afrotropical regions respectively, and one in the Palearctic Region (De Prins and De Prins 2005, 2011). Prior to this study, three species, Conopomorpha litchiella Bradley, 1986, Conopomorpha sinensis Bradley, 1986 and Conopomorpha cramerella (Snellen, 1904), were recorded in China. They are important fruit pests on litchi, longan and cacao in Fujian, Guangdong, Hainan, Hong Kong, Taiwan (Hwang et al. 1989, Huang et al. 1997, Robinson et al. 2001, Shapiro et al. 2008).

Meyrick (1880) described Epicephala based on the type species Epicephala colymbetella Meyrick, 1880. Species of this genus are extremely similar and difficult to distinguish. They usually have a fine or indistinct, curved, transverse silvery-metallic line before the apical area and a small round black dot in the apical area. The general structure is close to the genus Caloptilia Hübner, 1825, differing from it in the venation and in the peculiar resting posture: the Epicephala adult rests with its head appressed horizontally, the hind-part raises considerably and is seemingly supported by the hind legs, the fore and mid legs extended laterally and appressed to the horizontal surface (Meyrick 1880); the hind tibiae are bristly above, which also distinguishes the genus from Caloptilia. The wing pattern of Epicephala shows some resemblance to the genus Stomphastis Meyrick, 1912,but from this Epicephala can be separated by the wing venation and the very peculiar shape of the apo- and antapophyses in the female genitalia (Vári 1961).

Epicephala includes 40 described species: 18 in the Oriental Region, 15 in the Australian Region, six in the Afrotropical Region, and one in the Palearctic Region (Russian Far East). Two species were recorded to occur in China prior to this study: Epicephala venenata Meyrick, 1935 and Epicephala albifrons (Stainton, 1859) (De Prins and De Prins 2005, Kendrick 2005). Epicephala venenata occurs only in Taiwan, and the hostplant is unknown. Epicephala albifrons is widely distributed in Hong Kong, India, Indonesia, Sri Lanka, Thailand and Vietnam. The larvae of Epicephala albifrons are known to feed on Phyllanthus niruri Linn. (Euphorbiaceae).

In 2007, we discovered Conopomorpha flueggella Li, sp. n. and Epicephala relictella Kuznetzov, 1979 in the Baxian Mountain State Nature Reserves in Tianjin, China, whose larvae feed on Flueggea suffruticosa (Pall.) Baill. (Euphorbiaceae). Epicephala relictella is the only species of the genus distributed in the Palearctic Region, and is newly recorded for China. Its hostplant and biology were unknown previously.

## Results

### Conopomorpha flueggella

Li sp. n.

urn:lsid:zoobank.org:act:66017EDE-6984-4D06-B309-9414D0BE3C19

Figs 5–10 11 13 

#### Type material.

Holotype ♂ – **China,** [1] **Tianjin:** Baxian Mountain [40°11'03"N, 117°32'55"E], | Ji County, 600 m, 23.VII.2009, | Bingbing Hu reared [from fruit of Flueggea suffruticosa (Pall.) Baill.]. [2] Conopomorpha | *flueggella* | Li, sp. nov.Holotype ♂. Paratypes – 82 *♂♂*, 172 ♀♀, same data as for holotype except date and altitude: 19–24.VIII.2007, 10.V.–26.VII.2008, 16.V.–30.VIII.2009, 290–600 m; 1 *♂*, Limutai (40°11'17"N, 117°33'23"E), Ji County, 360 m, 24.VI.2009, coll. Bingbing Hu.

#### Diagnosis.

This species is similar to Conopomorpha litchiella, but distinguishable by the uniformly greyish brown to dark brown forewing with three pairs of stripes (more conspicuous when moths alive); the valva without protuberance on ventral margin distally and the saccus long linguiform in the male genitalia; the corpus bursae shorter than twice the length of the ductus bursae in the female genitalia; and the larva red-coloured. In Conopomorpha litchiella, the forewing is whitish yellow in distal portion; the valva has one large and one small protuberance on ventral margin distally, and the saccus is very short and small; the corpus bursae is twice as long as the ductus bursae; and the larva is yellowish green.

#### Description.

##### Adult

**Figures 5–10.**
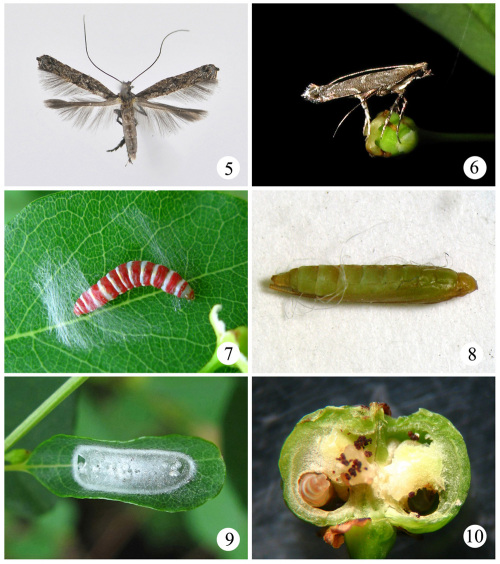
Life history of Conopomorpha flueggella. **5** adult, holotype, male **6** female moth resting on a female flower at night **7** mature larva weaving pupal cocoon on a host leaf **8** pupa **9** pupal cocoon on a host leaf **10** infested fruit.

(Figs 5–6). Wing expanse 8.0–15.5 mm. Head grey to greyish brown, frons greyish white. Compound eye dark brown. Labial palpus white, second segment with outer surface and distal tuft of ventral surface fuscous, third segment porrect or obliquely upward. Maxillary palpus greyish brown to dark brown. Antenna with scape greyish brown, flagellum brown to dark brown ringed with greyish white basally. Thorax and tegula dark brown. Forewing narrow, costal and dorsal margins nearly parallel; ground color greyish brown to dark brown; costal and dorsal margins with three oblique greyish white stripes respectively, first costal stripe from near middle extending obliquely to end of cell; dorsal margin with black speck at basal 1/3; bluish grey fascia with metallic reflection extending from near costal 5/6 to dorsum and along termen, respectively, between them set a large black spot; cilia pale greyish brown except fuscous apically. Hindwing and cilia greyish brown. Fore and mid legs brown; hind leg greyish white, distal half of tibia dark fuscous on outer surface. Abdomen grey, with first two segments shining white; ventral surface with five pairs of dark brown stripes along lateral sides.

##### Male genitalia

**Figures 11–14.**
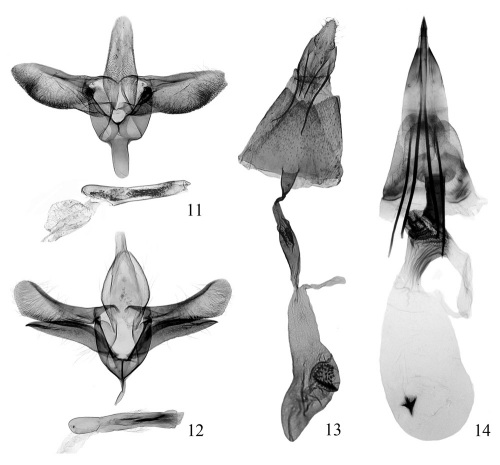
Genitalia of two gracillariidspecies. **11** Conopomorpha flueggella, male, paratype, slide No. BHY07239 **12** Epicephala relictella, male, slide No. BHY07296 **13** Conopomorpha flueggella, female, paratype, slide No. HBB09034 **14** Epicephala relictella, female, slide No. BHY08143.

(Fig. 11). Tegumen narrowed gradually to rounded caudal margin, with lateral side straight. Tuba analis indistinct. Valva broad, slightly longer than tegumen; costa nearly straight, basal half slightly sinuate, apex rounded; ventral margin of valva roundly protruded medially, densely with fine hairs; sacculus narrow and short, about 1/4 length of valva. Vinculum broad and short, nearly quadrate. Saccus long linguiform, about half length of tegumen, rounded at apex. Phallus tubular, nearly straight, as long as valva, medially with dense small spines inside.

##### Female genitalia

(Fig. 13). Papillae anales short and small, sparsely with setae. Apophysis anterioris thicker than and 1.6 × as long as apophysis posterioris. Antrum long funnel-shaped. Ductus bursae longer than apophysis anterioris, membranous except posterior 1/3 sclerotized and narrowed, medially expanded slightly and with longitudinal carinae. Corpus bursae membranous, prolonged pyriform, about 1.5 × as long as ductus bursae, with one side concave; signum large, rounded, situated at middle, covered with spines.

##### Egg.

Flat, elliptic, 0.3 mm in length and 0.2 mm in width. Transparent membrane in surface, irregular meshy stripe on egg shell. Milky white, semitransparent; straw yellow when close to hatch.

##### Larva

(Fig. 7). Young instar larva flat, yellowish white, semitransparent, segments distinct, with sparse setae, anterior end wider than posterior. Head capsule semicircular, brown; mandible strong, protruded like pincers. Mature larva 5.5–7.0 mm; head deep brown, anterior 1/2–2/3 of each segment on thorax and abdomen red, posterior 1/3–1/2 white. Body with sparse setae. Three pairs abdominal legs on segment 3, 4 and 5 respectively; anal leg protruded backward.

##### Pupa

(Fig. 8). 4.0–6.0 mm, fusiform. Greenish yellow in early pupal stage, changing gradually to yellowish brown, blackish brown before eclosion. A corniform cocoon breaker on forehead. Forelegs to third abdominal segment, midlegs to fourth abdominal segment, hindlegs to seventh or eighth abdominal segment, wings to fifth abdominal segment, antenna to or slightly exceeding end of abdomen.

##### Cocoon

(Fig. 9). 7.0–9.0 mm, white, flat elliptic, with some white grains attached on surface.

#### Host plant.

Euphorbiaceae: Flueggea suffruticosa (Pall.) Baill.

#### Life history.

Conopomorpha flueggella has two generations annually in Tianjin, China (Table 1). The larvae feed on the seeds of Flueggea suffruticosa (Fig. 10). Mature larvae quit the fruits before they are ripe and pupate on leaves or leaf litter. The pupal stage lasts from 9 to 12 days. Adults of the second generation hibernate. Adults occur from May to the first ten days of June, and from the last ten days of June to the first ten days of August. Adults can emerge during the whole day, but the peak occurs in the morning. The mating occurs usually in the morning. At night, the moths are actively drinking nectar and ovipositing. Adults come sometimes at light. A parasitic Ichneumonid species was reared from pupae collected on leaves of Flueggea suffruticosa in the field.

**Table 1.** Annual life history of Conopomorpha flueggella in Tianjin, China.

Months/Generations	1–4			5			6			7			8			9			10–12		
	F	M	L	F	M	L	F	M	L	F	M	L	F	M	L	F	M	L	F	M	L
Second generation	(+)	(+)	(+)	+	+	+	+														
First generation				●	●	●	●														
					–	–	–	–	–												
							□	□	□		□										
									+	+	+	+	+								
Second generation (hibernating)									●	●	●	●	●								
										–	–	–	–	–							
												□	□	□	□	□					
															(+)	(+)	(+)	(+)	(+)	(+)	(+)

● egg, ― larva, □ pupa, + adult, (+) adult hibernating.F: First ten days, M: Middle ten days, L: Last ten days.

#### Distribution.

China (Tianjin).

#### Etymology.

The species name is derived from the larval host plant, Flueggea.

### Epicephala relictella

Kuznetzov, 1979

Figs 12 14 15–20

Epicephala relictella Kuznetzov 1979: 854; Kuznetzov 1981: 179; De Prins and De Prins 2005: 181; Kawahara et al. 2010: 132.

#### Material examined.

**Russia:** Holotype ♂, – Southern Maritime Territory, Gornotayezhnaya Station, 12.VII.1978, coll. V. I. Kuznetzov [in Russian]. Paratypes – 2 *♂♂*, 1 ♀, same data as for holotype but dated 3.VII.1978. **China, Tianjin:** 20 ♂♂, 9 ♀♀, Mt. Jiulong, Ji County, 130–200 m, 9–28. VI.2004; Limutai, Ji County, 300 m, 11.VI.2004, coll. Houhun Li et al., 6 *♂♂*, 1 ♀, 24.VI.2009, coll. Bingbing Hu; 1 ♂, 2 ♀♀, Baxian Mountain, Ji County, 550 m, 15.VII.2007, coll. Mingfeng Cao & Bingbing Hu; 74 *♂♂*, 60 ♀♀, 290–480 m, 8.V.–1.VII.2008, 19.V.–30.VIII.2009, coll. Bingbing Hu; **Hebei Province:** 1 ♂, Shangsi, Xiaowutai, Wei County, 1200 m, 25.VII.2000, coll. Yanli Du & Zhendong Li; **Heilongjiang Province:** 1 ♀, Haerbin, 150 m, 22.VII.1997, coll. Houhun Li; **Gansu Province:** 1 ♂, 2 ♀♀, Bifenggou, Wenxian, 860 m, 10–12.VII.2005, coll. Haili Yu.

**Figures 15–20.**
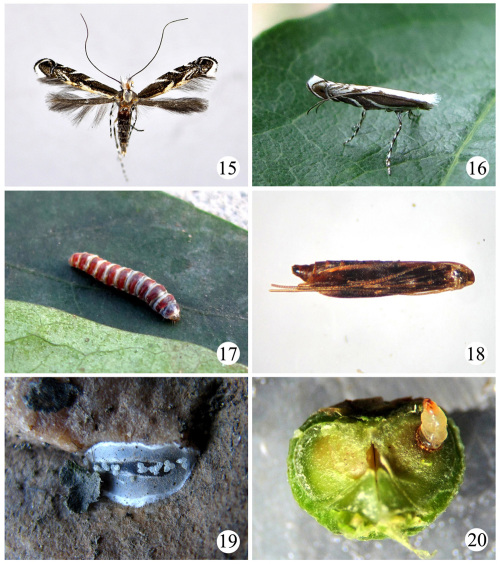
Life history of Epicephala relictella. **15** adult **16** moth resting on a leaf of host **17** mature larva resting on host leaf **18** pupa **19** pupal cocoon on stone nearby host **20** infested fruit.

#### Redescription.

##### Adult 

(Figs 15–16). Wing expanse 9.0–13.0 mm. Head white, tufted. Labial palpus white except outer surface grey. Antenna with scape pale grey dorsally, white ventrally; flagellum dark brown dorsally, copper-colored ventrally. Thorax white. Tegula and forewing greyish brown; white stripes at costal 2/5, 3/5, 4/5 and near apex as well as at dorsal 2/5 and 3/5 respectively, concentrated obliquely outward to 2/3 length and outside of cell, outmost one shortest, between first three stripes sometimes with short white strigulae; thin bluish white fascia with metallic reflection extending from costal 5/6 to dorsum; large black spot near apex; dorsal margin white tinged with ocherous yellow, longitudinally forming a broad band; termen dark brown; cilia white except fuscous distally from costal 5/6 along termen to before tornus, greyish brown along dorsal margin. Hindwing and cilia greyish brown. Fore and mid legs brown, hind leg greyish white, tibiae and tarsi with white rings. Abdomen greying brown on dorsal surface except first two segments grey; vental surface grey, with five pairs of oblique dark brown stripes along lateral sides.

##### Male genitalia

(Fig. 12). Tegumen broadly elliptic, caudal margin rounded. Tuba analis broad, distinct. Valva narrow, slightly longer than tegumen, expanded subapically, rounded at apex; costa sclerotized, gently concave; ventral margin nearly straight except basal 1/4 oblique, with dense fine hairs. Sacculus sclerotized, separated from valva, elongate lanceolate, about 4/5 length of valva; dosal margin gently arched, ventral margin slightly concave medially; distal portion longitudinally with sclerotized carina, apex spiculate. Vinculum broad, rounded anteriorly. Saccus slender, tapering, about 1/3 length of tegumen. Phallus tubular, straight, as long as valva, apex truncate; cornuti composed of dense small spines, compacted into one to three bundles.

##### Female genitalia

(Fig. 14). Ovipositor sclerotized to a strong spine, extensible. Apophysis very strong, apophysis posterioris slightly longer than apophysis anterioris. Lamella antevaginalis nearly trapezoid, caudal margin concave medially. Antrum strongly sclerotized, about half length of apophysis anterioris, oblique anteriorly. Ductus bursae thick and short, weakly sclerotized, slightly longer than antrum, expanded with irregular sclerotized carinae posteriorly, narrowed gradually towards corpus bursae, with sclerotized longitudinal pleats. Corpus bursae membranous, elongate elliptic, about same length as apophysis posterioris; signum small, coniform or stelliform, placed anteriorly.

##### Egg.

Oval, diameter about 0.15–0.20 mm. Surface smooth, shiny. Egg first yellowish white, nearly transparent, then becoming straw yellow before hatching.

##### Larva

(Fig. 17). Young instar larva very similar to that of Conopomorpha flueggella. Mature larva 5.0–6.5 mm; head capsule brownish yellow, median 2/3 of each segment on thorax and abdomen dark red, anterior and posterior ends white; thoracic segments slightly blue, abdominal segments with blue spots. Body with sparse white setae. Three pairs abdominal legs on segment 3, 4 and 5 respectively; anal leg protruded backward.

##### Pupa

(Fig. 18). 4.0–5.5 mm, fusiform. Greenish yellow in early pupal stage, changing gradually to dark brown. A corniform coccon breaker on forehead. Forelegs to third abdominal segment, midlegs to fourth abdominal segment, hindlegs to eighth abdominal segment, wings to fifth abdominal segment, antenna obviously exceeding end of abdomen.

##### Cocoon

(Fig. 19). 6.0–8.0 mm; white, flat elliptic, with some white grains attached on surface.

#### Host plant.

Euphorbiaceae: Flueggea suffruticosa (Pall.) Baill., recorded for the first time herein.

#### Life history.

Epicephala relictella has one generation annually in Tianjin, China (Table 2). The larvae feed on the seeds of Flueggea suffruticosa (Fig. 20). The larval stage is completed within one fruit. When completing larval development, the mature larvae quit the fruits and pupate on the leaves, and overwinter under leaf litter or stones.

Adults appear from June to July. They can emerge during the whole day, but the peak occurs in the morning. The moths are most active at night, drinking nectar and ovipositing. During the daytime they rest on leaves or branches. Adult longevity is 3–10 days, but adults generally live for 5–7 days. Adults hardly come to light.

#### Distribution.

China (Tianjin, Hebei, Heilongjiang, Gansu), Korea, Russia.

**Table 2.** Annual life history of Epicephala relictella in Tianjin, China.

MonthsGeneration	1–5			6			7			8			9			10–12		
	F	M	L	F	M	L	F	M	L	F	M	L	F	M	L	F	M	L
First generation	(□)	(□)	(□)	(□)	(□)	(□)	(□)											
				+	+	+	+	+										
				●	●		●	●	●									
						–	–	–	–	–	–	–	–	–				
									(□)	(□)	(□)	(□)	(□)	(□)	(□)	(□)	(□)	(□)

● egg, – larva, □ pupa, (□) pupa through the winter, + adultF: First ten days, M: Middle ten days, L: Last ten days.

**Table 3.** Life history comparisons of Conopomorpha flueggella and Epicephala relictella

	Characteristics	Conopomorpha flueggella	Epicephala relictella
Similarities	Feeding habits	Seed parasite	
	Pupation site	Boring an exit hole to escape from the fruit to pupate on the leaves or litter	
	Mating site	On the leaves of host	
	Do adults feed ?	Yes	
Differences	Flight period	In early May, slightly earlier than flowering season	In early June, keeping pace with early fruiting season
	Overwintering	Adult	Pupa
	Generation	Two generations annually	One generation annually
	Phototaxy	Feeble	Hardly any

## Discussion

Calybites securinella (Ermolaev, 1986) was the only species in Gracillariidae known to be associated with Flueggea suffruticosa. It occurs in Russia (Primorye) and Korea (Ermolaev 1986, Kawahara et al. 2010). Associations of the gracillariidmothswith fruits or seeds of different euphorb genera, including Flueggea, are known for Epicephala (Kawakita and Kato 2009). Conopomorpha flueggella is another seed-feeder, similar to several species of the genus that feed on fruits or seeds of other plant families (Bradley 1986).

Similar to Conopomorpha flueggella, Epicephala relictella also feeds on the seeds of Flueggea suffruticosa. They are very similar in morphology and biology, and hard to distinguish. Table 3 compares the life histories of the two species.

Most gracillariid species are leaf-miners, and the seed-parasitic habit is infrequent. Epicephala is noteworthy for its obligate pollination habit, which involves a mutualistic relationship with trees of Euphorbiaceae (Kawakita 2010). However, Epicephala relictella is not pollinating its host.

## Supplementary Material

XML Treatment for Conopomorpha flueggella

XML Treatment for Epicephala relictella
